# An evaluation of emerging vaccines for childhood pneumococcal pneumonia

**DOI:** 10.1186/1471-2458-11-S3-S26

**Published:** 2011-04-13

**Authors:** Julia Webster, Evropi Theodoratou, Harish Nair, Ang Choon Seong, Lina Zgaga, Tanvir Huda, Hope L Johnson, Shabir Madhi, Craig Rubens, Jian Shayne F Zhang, Shams El Arifeen, Ryoko Krause, Troy A Jacobs, Abdullah W Brooks, Harry Campbell, Igor Rudan

**Affiliations:** 1 Centre for Population Health Sciences, Global Health Academy, The University of Edinburgh, UK; 2 Public Health Foundation of India, New Delhi, India; 3 International Centre for Diarrhoeal Disease Research, Bangladesh, Dhaka, Bangladesh; 4 Department of International Health, Bloomberg School of Public Health, Johns Hopkins University, Baltimore, MD, USA; 5 Department of Science and Technology/National Research Foundation: Vaccine Preventable Diseases & Medical Research Council Respiratory and Meningeal Pathogens Research Unit, University of the Witwatersrand, South Africa; 6 Center for Childhood Infections and Prematurity Research, Seattle Children's Met Park West, Seattle, USA; 7 International Federation of Pharmaceutical Manufacturers & Associations, Geneva, Switzerland; 8 USAID, GH/HIDN/MCH, Washington DC, USA; 9 Croatian Centre for Global Health, University of Split Medical School, Croatia

## Abstract

**Background:**

Pneumonia is the leading cause of child mortality worldwide. *Streptococcus pneumoniae* (SP) or *pneumococcus* is estimated to cause 821,000 child deaths each year. It has over 90 serotypes, of which 7 to 13 serotypes are included in current formulations of pneumococcal conjugate vaccines that are efficacious in young children. To further reduce the burden from SP pneumonia, a vaccine is required that could protect children from a greater diversity of serotypes. Two different types of vaccines against pneumococcal pneumonia are currently at varying stages of development: a multivalent pneumococcal conjugate vaccine covering additional SP serotypes; and a conserved common pneumococcal protein antigen (PPA) vaccine offering protection for all serotypes.

**Methods:**

We used a modified CHNRI methodology for setting priorities in health research investments. This was done in two stages. In Stage I, we systematically reviewed the literature related to emerging SP vaccines relevant to several criteria of interest: answerability; efficacy and effectiveness; cost of development, production and implementation; deliverability, affordability and sustainability; maximum potential for disease burden reduction; acceptability to the end users and health workers; and effect on equity. In Stage II, we conducted an expert opinion exercise by inviting 20 experts (leading basic scientists, international public health researchers, international policy makers and representatives of pharmaceutical companies). The policy makers and industry representatives accepted our invitation on the condition of anonymity, due to sensitive nature of their involvement in such exercises. They answered questions from CHNRI framework and their “collective optimism” towards each criterion was documented on a scale from 0 to 100%.

**Results:**

The experts expressed very high level of optimism (over 80%) that low-cost polysaccharide conjugate SP vaccines would satisfy each of the 9 relevant CHNRI criteria. The median potential effectiveness of conjugate SP vaccines in reduction of overall childhood pneumonia mortality was predicted to be about 25% (interquartile range 20-38%, min. 15%, max 45%). For low cost, cross-protective common protein vaccines for SP the experts expressed concerns over answerability (72%) and the level of development costs (50%), while the scores for all other criteria were over 80%. The median potential effectiveness of common protein vaccines in reduction of overall childhood pneumonia mortality was predicted to be about 30% (interquartile range 26-40%, min. 20%, max 45%).

**Conclusions:**

Improved SP vaccines are a very promising investment that could substantially contribute to reduction of child mortality world-wide.

## Background

Pneumonia is the leading single cause of mortality in children under the age of 5 years worldwide [1]. Many of these pneumonia related deaths are vaccine preventable. The global burden of disease of pneumococcal pneumonia is difficult to determine, particularly in developing countries with limited surveillance facilities and routine health and health services data [2]. However a recent systematic review of disease burden in children under the age of five reported that in 2000, an estimated 14.5 million episodes of severe pneumococcal disease occurred, causing 821,000 deaths [3]. Of these, 88,000 deaths occurred among HIV positive children, of which 61% were in 10 countries all located in Africa and Asia [3].

*Streptococcus pneumoniae* (SP) has at least 92 serotypes. The most frequently used vaccine is the seven valent, protein conjugate vaccine (Prevnar), protecting against the serotypes that are most common in Northern America [2]. These serotypes account for only approximately 39% of the invasive disease-causing serotypes in Africa, 48% in Asia and 53.4% in Latin America and the Caribbean, due to the biological diversity of *S.pneumoniae*[4-6]. Additionally, replacement disease [6] from non-vaccine serotypes has had varying effects in different settings, including reports of emerging drug resistance [1,2], on the effect of pneumococcal conjugate vaccine (PCV) against overall invasive pneumococcal disease, though the overall rates of antibiotic resistant pneumococci have not increased following the introduction of PCV. Current ten- and thirteen-valent pneumococcal conjugate vaccines that have obtained regulatory approval worldwide contain over 70% of the estimated invasive pneumococcal disease that is caused globally. Eighty percent of global disease is caused by 17 serotypes (95% CI 14-21) [7], and different serotypes predominate in varying geographical regions [2], and differ in prevalence among important clinical syndromes [8].

In order to prevent pneumonia infection due to any serotype of *S.pneumoniae* there are two main vaccine development strategies:

• a serotype-based PCV covering as high a proportion as possible of all disease-causing serotypes. At the recent International Symposium on Pneumococci and Pneumococcal Diseases, Merck discussed further increasing vaccine valency by developing a 15-valent vaccine (Johnson H, personal communication). PATH is currently sponsoring emerging manufacturers to develop at least one geographically tailored vaccine that will meet the pneumococcal Advance Market Commitment (AMC). In addition, with the AMC other vaccine manufacturers may also be developing multi-valent pneumococcal conjugate vaccines with support from PATH (as announced in the 2010 ISPPD);

• a pan-serotype protective vaccine using conserved common pneumococcal protein antigens (PPA) (combinations of these two strategies are also under consideration). Potential common protein vaccines are in phase 1 clinical trials, with other vaccine candidates in the pre-clinical stages.

The aim of this briefing paper is to present the evidence regarding key issues surrounding the first two vaccine development strategies and assess the level of collective optimism among international experts concerning the level of investment priority they feel is justified. The paper is presented as part of a series of papers, each in turn focusing on different emerging vaccines and other interventions against pneumonia.

## Methods

We used a modified Child Health and Nutrition Research Initiative (CHNRI) methodology for setting priorities in health research investments. The methodology has been described in detail [9-13] and implemented in a variety of settings [14-18]. Briefly, the method uses a set of pre-defined criteria and collects expert opinion of all stakeholders on the risks and benefits associated with investing in existing and/ or new interventions.

### CHNRI exercise – stage I: Identification and selection of studies

**Figure 1:**
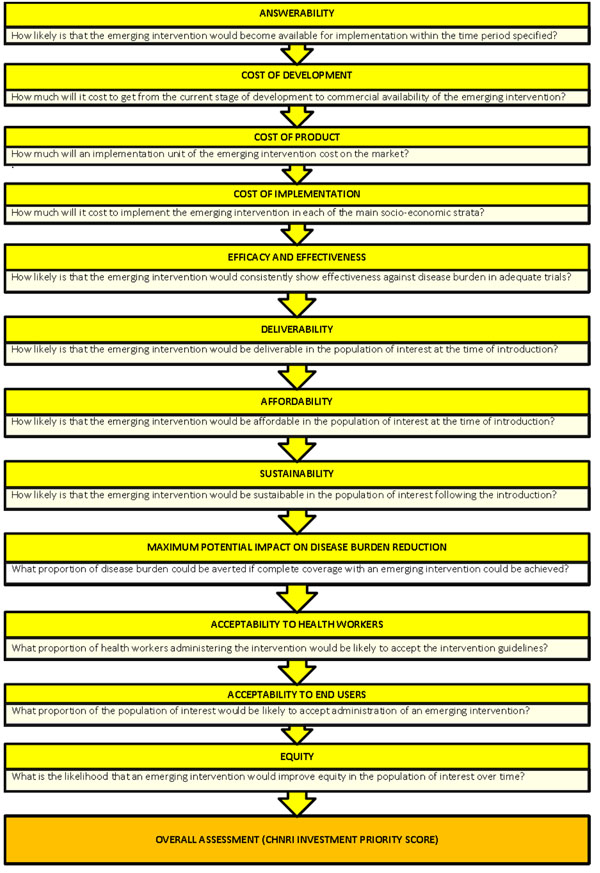
A summary of Stage I of the CHNRI process of an evaluation of emerging intervention (a systematic review of the key CHNRI criteria)

A literature search was conducted for each of the 9 CHNRI criteria (Figure 1): answerability, cost of development, cost of product, cost of implementation, efficacy and effectiveness, maximum potentail for disease burden reduction, deliverability, affordability and sustainability, acceptability to health workers, acceptability to the end users and effect on equity [19]. Details about the search strategies are presented in Additional File 1. The databases Ovid MEDLINE (1950 to 2009), EMBASE (1980 to 2009) and GLOBAL HEALTH (1973 to 2009) were searched. To avoid database bias and to identify studies from developing countries LILACS (Latin American and Caribbean Health Sciences) and IndMed (Indian Medlars Centre) were also searched but did not yield any additional citations. Additionally a grey literature database (SIGLE) and Cochrane central register for controlled trials were also searched but again did not yield any additional results. Searches were conducted, and subsequently updated between the 16^th^ March and 24^th^ May 2009, to ensure the most recently published material was included. In order to ensure completeness, we also conducted hand searching of online journals, scanned the reference list of identified citations, and perused literature available on the websites of pharmaceutical companies - Wyeth (later acquired by Pfizer), GlaxoSmithKline and Intercell and international agencies (GAVI, WHO, UNICEF and Pneumo ADIP)

Eligible studies were selected according to the pre-determined inclusion criteria. Included studies: (i) were publications from developing countries and (ii) investigated the effect, or distribution, coverage and delivery of multivalent pneumococcal conjugate vaccines and/ or cross-protective common protein vaccines, including indirect effects of immunisation; or (iii) described the global burden of disease of pneumococcal pneumonia in children under 5; or (iv) were historical papers for comparison with the most recently published material. Studies not eligible for inclusion were those: (i) examining the effect of pneumococcal polysaccharide vaccine; (ii) describing the global burden of disease in adults; (iii) presenting delivery strategies for developed countries; and (iv) describing the burden of only pneumococcal otitis media and meningitis. Data from developed countries were used, when data from developing where not available

### CHNRI exercise – stage II: An expert opinion exercise

**Figure 2:**
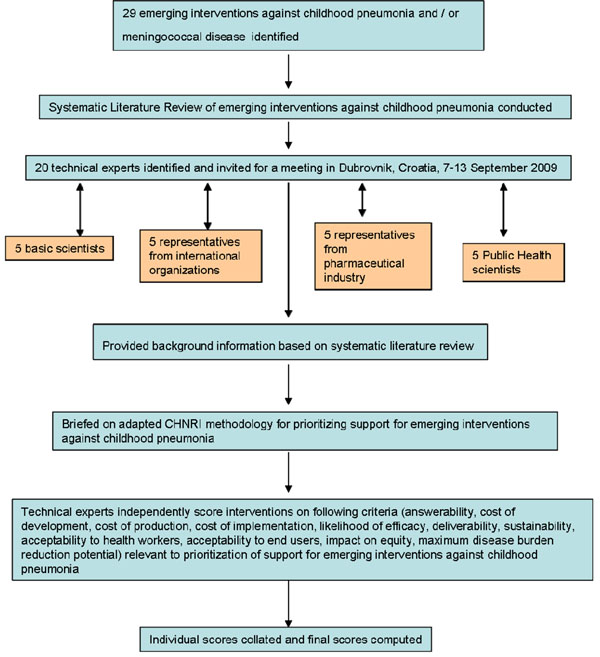
A summary of Stage II of the CHNRI process of an evaluation of emerging intervention (an expert opinion exercise using the CHNRI criteria)

We shared the initial review of the literature with 20 experts that met during September 7-13, 2009 in Dubrovnik, Croatia, to conduct the 2^nd^ stage of CHNRI expert opinion exercise. They were chosen based on their outstanding track record in childhood pneumonia or pneumococcal disease research. We initially offered participation to those experts with the greatest impact of publications in their area of expertise over the past 5 years (for basic researchers and international public health researchers), or to those that were affiliated with the largest pharmaceutical company in terms of vaccination programme or international agency in terms of their annual budget. For those who declined to participate (about 20%) replacements were found using the same criteria. The process of second-stage CHNRI is shown in Figure 2. All invited experts discussed the evidence provided in CHNRI stage I, and then answered questions from CHNRI framework (see Additional File 2). Their answers could have been “Yes” (1 point), “No” (0 points), “Neither Yes nor No” (0.5 points) or “Don’t know” (blank). Their “collective optimism” towards each criterion was documented on a scale from 0 to 100%. The interpretation of this metric for each criterion is simple: it is calculated as the number of points that each evaluated type of emerging SP vaccine received from 20 experts (based on their responses to questions from CHNRI framework), divided by the maximum possible number of points (if all answers from all experts are “Yes”).

## Results

Details of the results of the literature search are presented in Additional File 1 For SP vaccines, 141 abstracts were considered and 14 papers were selected for inclusion. Similarly, for common protein vaccines, 459 abstracts were considered and 7 papers were selected for inclusion. Additional searches for deliverability, equity and Global Burden of Disease were conducted and 506 were selected for abstract screening, 21 of which were included in the review. In the following paragraphs, the results of the literature search for each criterion will be presented alongside a description of how well the particular emerging intervention scored in the CHNRI exercise.

### Answerability

This was defined as achievement of a research goal of the production of an effective novel vaccine that can be fitted into the routine Expanded Programme of Immunisation (EPI) schedule within in a time frame of 10 years.

#### Pneumococcal Conjugate Vaccine

PCVs are generally well tolerated and safe, including when co-administered with other childhood vaccines. They are formulated by conjugating multiple serotype-specific capsular polysaccharide epitopes to a carrier protein [20]. PCV-7 and -13 formulations are conjugated to cross reactive material 197 protein (CRM197), which is a mutant diphtheria toxoid molecule. Most serotypes in the PCV10 formulation are conjugated to protein D derived from non-typeable *Haemophilus influenzae* (NTHi) [21]. PCVs are immunogenic in children under two years of age [20], whereas the polysaccharide vaccine is not. The PCV7 was first licensed in February 2000 and higher valency (10- and 13-) PCVs formulations have been licensed since 2009. However, the possibility of adding further serotypes appears to be limited, mainly because the development cost is high and also because the conjugation process and retaining of immunogenicity for each of the included serotypes (which are not responsible for a large proportion of disease) is complicated. In addition, there is evidence showing a dampening of the immunogenencity to select common serotypes in children vaccinated with PCV13 compared to those vaccinated with PCV-7 [22,23]. This is possibly related to the development of tolerance to vaccine components or other interference by inclusion of multiple serotypes.

Some serotypes of *S.pneumoniae* more commonly cause disease, and the prevalent causative serotypes also vary geographically [24,25]. The current PCV7 covers those serotypes found most commonly in North America, whereas PCV10 and 13 also include some serotypes that are common in Africa as well as Asia. More recently, consensus is being build over a set of 7 serotypes that are common globally and a vaccine developed containing these serotypes could provide serotype coverage closer to a 10 & 13 valent vaccine [6]. However, as 10 & 13 valent vaccines are already available, manufactures have less incentive to develop such vaccines. A geographically tailored vaccine covering fewer serotypes, but specifically targeting those most prevalent in a given area could also be an option. The cost of this makes it an unlikely option, though. The issue of serotype “replacement colonisation” would still remain. However, the debate on serotype replacement has been complicated, in some instances, by studies that have failed to distinguish serotype replacement of colonising bacteria in the nasopharynx from replacement of those serotypes associated with invasive disease, and whether these are actually the same [26].

**Figure 3:**
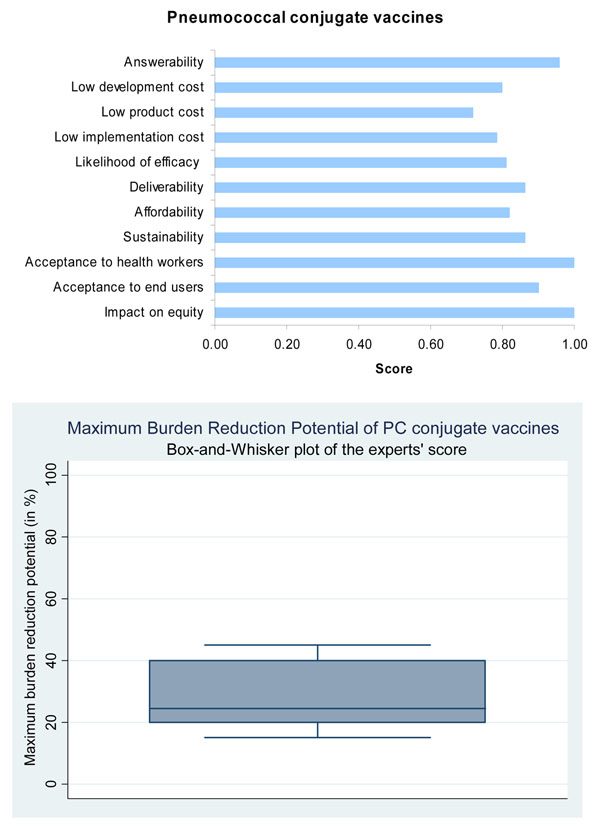
The results of Stage II CHNRI process – an expert opinion exercise assessing the potential usefulness of investment in low-cost pneumococcal conjugate vaccines

Presented with this evidence, the panel of experts expressed a very high level of optimism (over 80%) on the ability of PCV to satisfy the criterion of answerability (Figure 3).

#### Common protein vaccine

In 1991, one of the first papers regarding a monoclonal antibody against pneumococcal surface protein A (PspA) was published [27]. It was shown to protect mice from fatal pneumococcal infection, and it was thought that it would be able to elicit a cross-protective response across heterotypic pneumococcal strains [28]. There has been ongoing research to identify other PPAs which either individually or in combination may provide cross-protection across different strains and serotypes of pneumococci [29].

Recently, novel antigens have been identified which take advantage of the complete bacterial genome sequence [30-32]. In late 2007 the lead vaccine candidates serine/threonine protein kinase (StkP) and the protein required for cell wall separation of group B streptococcus (PcsB) were identified [33]. These were found to be greater than 99.5% conserved among clinical isolates and also cross-protective [27]. The antigens are immunogenic in both elderly and young children, the serotype-independent expression will combat varying strains and serotype distribution of pneumococci and could potentially limit the emerging importance of non-vaccine strains and serotypes of pneumococci [27]. There are other common protein antigen vaccine candidates in pre-clinical trials.

**Figure 4:**
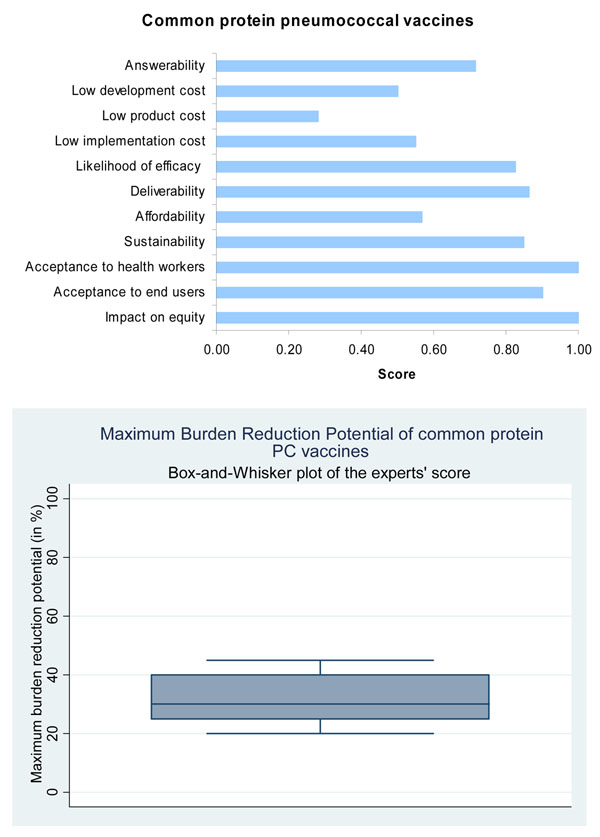
The results of Stage II CHNRI process – an expert opinion exercise assessing the potential usefulness of investment in low-cost common protein pneumococcal vaccines

Based on this evidence, the panel expressed concerns over the ability of the pneumococcal protein vaccine (PPV) to satisfy the answerability criterion (scored only 72%) when compared to the very high score (over 95%) for PCV (Figure 4).

### Efficacy - The impact of the vaccines under ideal conditions

#### Pneumococcal conjugate vaccine

**Figure 5:**
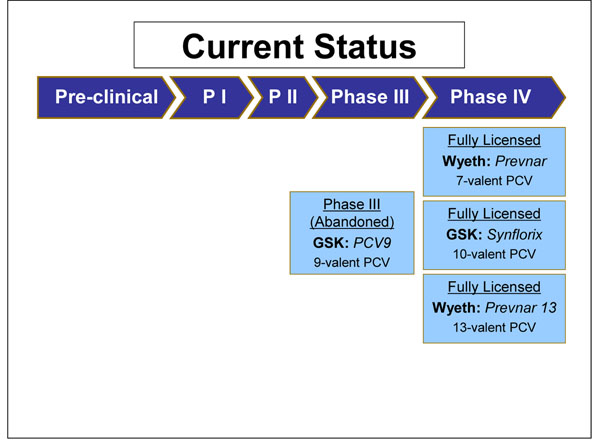
The current status of the research into SP vaccines, as of September 2009 (see Additional File 3 for details about the clinical trials phases)

PCV7 has completed all clinical trial stages (Figure 5**)**. PCV7 is 82-97% efficacious against invasive pneumococcal disease caused by vaccine serotypes, 90% efficacious against vaccine-serotype specific pneumococcal pneumonia and 57% efficacious against pneumococcal acute otitis media caused by vaccine serotypes [20].

PCV9 has also completed all clinical trial stages. However, it has not been developed further in favour of an expanded 13-valent formulation (Figure 5). In a clinical trial conducted in the Gambia the efficacy of PCV9 was 77% against invasive pneumococcal disease (IPD) caused by vaccine serotypes, 50% against disease caused by all serotypes, 15% against all-cause admissions and 16% against all-cause childhood mortality [34]. This is an important study as it is the largest of its kind to be conducted in a developing country (with 17,436 children participating in the study).

PCV10’s immunogenicity, safety and reactogenicity profile is comparable to PCV7 [35]. Co-administration studies have demonstrated its compatibility with major childhood vaccines [36]. A phase III clinical trial found that one month after dose 3, the percentage of subjects with adequate antibody concentrations against each of the pneumococcal serotypes was at least 96.6%, except serotype 6B which was 79.3%, and those with sufficient opsonophagocytic activity against each serotype was 98.0% [37]. In March 2009 GlaxoSmithKline received European Commission authorisation for Synflorix™ - their 10-valent PCV [21] - and received WHO prequalification, a pre-requisite for supply to GAVI-eligible countries, in November 2009 (Figure 5) [21].

There is limited published material regarding PCV13. Phase I trials have found PCV13 to be more immunogenic than the currently available 23vPS for most of the shared serotypes in the two vaccines, and it is generally well tolerated [38,39]. There are ongoing phase II and III trials (Figure 5) [40]. In July 2009 Pfizer announced that the Chilean Ministry of Health has become the first government agency to approve Prevenar 13* Valent [41]. They were granted European marketing authorisation for Prevenar 13* by the European Commission in December 2009 [42] and by the US Food and Drug Administration [43].

Based on this evidence, the panel was optimistic that all PCV vaccines would have a high likelihood of being efficacious (Figure 3).

#### Common protein vaccine

**Figure 6:**
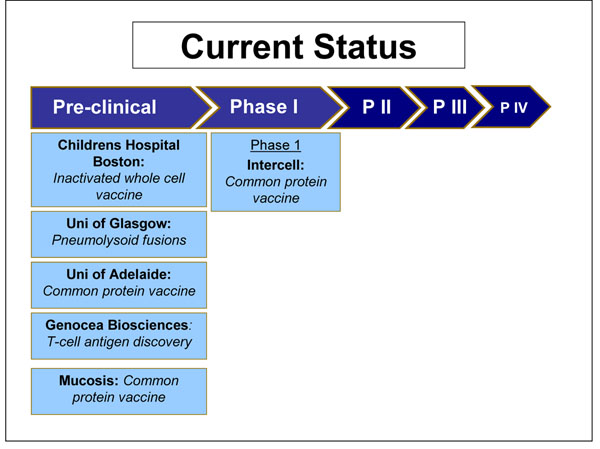
The current status of the research into common protein SP vaccines, as of September 2009 (see Additional File 3 for details about the clinical trials phases

In April 2009 Intercell announced that they are beginning a phase I clinical trial of a vaccine containing three conserved surface proteins StkP, PcsB and PSaA [44]. The vaccine formulation is currently being evaluated for immunogenicity in different populations (Figure 6).

Mucosis and the University of Adelaide are currently working on a common protein vaccine, but these are in the pre-clinical stages and there is no published information. Genocea Biosciences is working on T-cell antigen discovery, again in the pre-clinical stages. Children’s Hospital Boston is developing an inactivated whole cell vaccine for phase 1 clinical trials. The aim is that it would be low cost to manufacture, would require no refrigeration and could be given orally or intranasally. The University of Glasgow is developing pneumolysoid fusions which will act as an antigen and adjuvant for carried protein. It would provoke an immune response after a single mucosal immunisation, and very small amounts of protein would be required (Figure 6) [45]. In this case too, the panel felt that PPV would have a high likelihood of efficacy (Figure 4).

### Effectiveness - The impact of these vaccines in the population

#### Pneumococcal conjugate vaccine

Introduction of PCV7 in America led to a reduction in incidence of IPD of 69% in children under 1 year, 68% in children aged 1-2 years, 44% in children aged 2-3 years, and no reduction was seen in those aged 3-4 years [20]. PCV7 has diminished hospitalisation rates for all-cause pneumonia in young children by almost 40% in America [46].

Oosterhuis-Kafeja and colleagues estimated that the maximal achievable levels of theoretical serotype coverage of PCV 7 is 88.7% in North America and Australia, and 77.6% in Europe, where serotypes 1 and 8 are more prevalent [20]. They estimated though that the theoretical coverage of PCV 7 is lower in the developing world - 67.3% in Africa, 63.4% in Latin America and 43.1% in Asia, as serotypes 1 and 5 are highly prevalent in these regions. However, a recent study encompassing data from 22 studies including 11,181 serotyped isolates from children with invasive pneumococcal disease in Africa concluded that the coverage of PSV 7 in Africa was only 49%, whereas the coverage of a 10-valent vaccine, including types 1, 5 and 7F, would cover approximately 72% of invasive isolates from children, by offering coverage against the additional serotypes 1, 5 and 7F [47]. .

PCV 9 was found to be 77-83% effective against IPD by vaccine serotypes, and 36-50% protective against disease by all serotypes in HIV-uninfected children [25,38,39]. HIV is major risk factor for pneumococcal disease. Klugman and colleagues found that efficacy declined from 65% to 38.8% in HIV positive children 6.2 years following immunisation with PCV9 [48]. A high efficacy of 77.8% (95% CI 34.4, 92.5%) against vaccine serotypes was maintained in non-infected children, however the overall efficacy against IPD due to any serotype was only 35% (-30.6, 67.7%) [49]. Although PCV9 is effective in HIV positive children, the immunogenicity levels, persistence of antibodies and efficacy was lower compared to HIV non-infected children. Nevertheless as HIV infected children have a 40 fold greater burden of pneumococcal disease, despite lower vaccine efficacy, the absolute burden of IPD prevented was 18 fold greater in HIV infected children compared to HIV non-infected children [49,50].

Indirect immunity is protection in those who have not been vaccinated, due to the reduced risk of pneumococcus acquisition in vaccinated children, and interrupted transmission thereof to other members of society. A study in USA found that PCV 7 prevented twice as many cases through indirect protection compared to the direct effect of the vaccine in preventing IPD [51]. Colonisation of the nasopharynx is a pre-requisite to developing pneumococcal disease, although the predictors of who will develop disease following colonisation are less well known [20]. PCV7 and PCV9 have both been shown to reduce nasopharyngeal acquisition of pneumococcus by some vaccine serotypes. Siblings of children vaccinated with PCV9 were also less likely to become colonised by the vaccine serotypes [20]. A recent American study found the contribution of indirect effects on IPD to be around 20% of the total benefit in children aged less than 5 [52].

A significant challenge of PCV vaccination targeting only select serotypes is the potential for replacement colonisation and disease occurring from non-vaccine serotypes [19]. The long term effect of replacement colonisation remains unclear, with differing experience in Alaskan native, US and UK general populations.

Based on this evidence, the panel predicted the median potential effectiveness of SP vaccines towards reduction of overall pneumonia mortality would be about 25% (interquartile range 20-38%, min. 15%, max 45%) (Figure 3).

#### Common protein vaccine

These vaccine candidates are in early trial stages, therefore no information is available regarding effectiveness in the population. It is thought a vaccine will induce herd immunity as animal models have found that select protein vaccines reduce the risk pneumococcal colonisation [53]. It is also though that a protein vaccine will be immunogenic in young children [53].

The panel predicted that the median potential effectiveness of PPV in reducing overall pneumonia mortality would be about 30% (interquartile range 26-40%, min. 20%, max 45%) (Figure 4).

### Cost of development and implementation

Cost and securing sustainable production capacity are major factors determining the deliverability of a vaccine. In the case of a pneumococcal vaccine an “advanced market commitment” (AMC) pilot has been established. An AMC provides a demand led approach by stimulating private investment in vaccine research and development, and increasing manufacturing capacity for vaccines which primarily address diseases of developing countries [54]. AMC donors guarantee the price of specific vaccines, aiming to reduce the time delay between the introduction of new vaccines into developed and developing countries. Through a legally binding contract, AMC participating companies commit to continuously supply the vaccines at lower and sustainable price to GAVI countries for a 10 year period [6,54].

While the panel was optimistic about the development of a low cost PCV, it expressed concern over the ability to develop a PPV with similarly low development costs (Figure 3 and Figure 4).

### Deliverability

It has been demonstrated that adequate infrastructure in the form of cold chain equipment, functioning health systems reinforced by refresher training of health workers, ongoing monitoring and periodic evaluations of vaccine coverage, surveillance systems to capture adverse events following immunization, and activities to generate high levels of awareness in the community are the keys to the successful deliverability of any new vaccine [55]. The deliverability of such a vaccine is enhanced if it can be integrated into the existing Expanded Programme of Immunization (EPI) schedules [56].

#### Pneumococcal conjugate vaccine

Since PCV 7 does not tolerate freezing, it should be stored at 2-8°C, and therefore requires a cold chain, similar to the current EPI vaccines [52]. Other PCVs currently in production are likely to have similar cold chain requirements, as they use a similar vaccine technology. PCV -7, -10 and -13 fit in to the current EPI schedule, and can be given at 6, 10 and 14 weeks. Although PCV 7 is safe to be co-administered alongside other vaccinations, an alternative body site is preferable [52]. In stage II of the modified CHNRI exercise, the experts were highly optimistic regarding the deliverability of PCV7 and thus scored it high on deliverability, with CHNRI score for this criterion greater than 80% (Figure 3).

#### Common protein vaccine

The specific delivery requirements of a common protein vaccine are unknown, as the trials are in very early stages. If other protein vaccines are used for comparison it is likely the vaccine will require refrigeration. The panel was optimistic regarding the deliverability of this vaccine, again with CHNRI score greater than 80% (Figure 4).

### Global burden of disease and disease burden reduction

#### Pneumococcal conjugate vaccine

Immunisation is the most effective method available to reduce morbidity and mortality from infectious diseases [2]. After introduction of Hib vaccine in Kenya, the prevalence of disease fell by 88% in three years [57]. This shows the dramatic impact an effective vaccine can have on disease burden, even in a developing country setting.

The underlying aim behind SP vaccine development approaches is that they will prevent infection by *S.pneumoniae*. It will not however protect neonates or children under 6 weeks of age if delivered within the existing child EPI schedule. Any indirect effects of the vaccine may have protective effect in this age group. Neonates and young infants are at risk of certain bacterial infections, including pneumococcal disease, but the incidence has not been clearly defined [58]. Between age 6 and 24 months is when the incidence of disease is at its highest [59]. In an American study comparing rates of pneumococcal infection before and after the introduction of PCV7, it was found that in infants aged 0 to 60 days the rate of IPD decreased from 7.3 per 100.000 live births to 4.2 per 100.000 live births [58]. This suggests that neonates and infants currently too young to receive PCV7, along with non-immunised members of the community, are benefiting from indirect protection [58]. Conjugate vaccines may even be able to induce herd immunity in situations where coverage is significantly incomplete, and fewer than the recommended number of doses have been administered [60]. Major problems encountered when treating *Streptococcus pneumoniae* infection especially in least developed countries is poor access to curative health-care and antibiotic resistance [59], therefore prevention with a vaccine is better than trying to cure the disease.

The potential disease burden reduction with PCV7 has not been maximised as it has yet to be distributed throughout most of Africa and Asia. PCV7 covers approximately 40-60% of the serotype distribution in Africa and Asia, where the majority of child deaths occur. If delivered at high immunization coverage levels, it has the potential to reduce deaths by approximately 50% - not including (positive and negative) indirect effects - which would save around 400,000 lives per year (assuming 100% of vaccine efficacy). Distributing PCV10, with higher disease coverage of 60 to 80%, has the capacity to increase that number to around 550,000, again not including (positive and negative) indirect effects.

Introducing a new vaccine can potentially also enhance delivery of existing vaccines [2] and increase coverage and uptake of vaccines generally [61]. This effect would contribute to a reduction in the burden of all vaccine preventable diseases but the reduction depends upon an array of systems issues that occur whether service delivery occurs in health facility, by outreach, or in the community.

#### Common protein vaccine

It is estimated that there are over 800,000 deaths from SP per year in children under age 5. Therefore, if the new PPV would indeed be 100% effective against all pneumococci world-wide, then it would have the potential to reduce disease burden by 100%, thus preventing avoiding 800,000 deaths per year. However, the assumptions are 100% immunization coverage (or enough to induce indirect protection) and quality control of the delivery of this vaccine in all settings. There are large problems with delivery in the most remote and poor settings, ranging from the breakdown of the cold chain to inadequate administration of the vaccine. Even when a vaccine attains high coverage, the last to be reached are often in the poorest areas which house a higher proportion of the disease burden, and children less than 6 weeks old only gain protection through herd immunity. In reality, the achievable disease burden reduction in children under age 5 would surely be less than that.

The expert group felt that both the pneumococcal conjugate vaccines and the common protein vaccines had high median potential effectiveness for reduction of pneumonia mortality (25%; interquartile range 20-38% and min. 15%, max 45%; and 30%; interquartile range 26-40% and min. 20%, max 45%, respectively) (Figures 3 and 4).

### Acceptability and equity

#### Pneumococcal conjugate vaccine

The distribution of communicable diseases globally highlights the inequity amongst the various population groups. Communicable diseases account for 68% of disease burden in Africa but only 7% in developed countries [54]. If this gap were reduced, much of the global difference in life expectancy and mortality would disappear. Even within a country, it is the poor and vulnerable who have reduced access to heath care and who experience a higher burden of disease [62]. Cunha and colleagues have shown that it is the children from low socio-economic strata living in developing countries who appear to be at the highest risk for acute lower respiratory tract infections [63]. Victora and colleagues have demonstrated an inverse relationship between social class and maternal education with the risk of developing pneumonia [64]. The economic consequences of pneumonia, including cost to family and the resulting disability, the economic pressure on developing governments and on struggling health systems lead to a cycle of poverty, further widening the gap of inequity [2].

The panel was optimistic that a highly effective conjugate vaccine against the pneumococcus would have a profound impact on decreasing child health inequity and would be accepted by the end-users and the health workers, with a score for each of these criteria greater than 90% (Figure 3).

#### Common protein vaccine

The panel was optimistic that a highly effective common protein vaccine against the pneumococcus would have a profound impact on decreasing child health inequity and would be accepted by the end-users and the health workers, with a score greater than 90%, and these scores were the same as for PCV (Figure 4).

## Discussion

The literature review summarized in this paper presents the available evidence required for making an informed decision on emerging pneumococcal vaccines to set research priorities. The score of both PCV and PPV against the criteria is the collective optimism of a panel of experts drawn from varying backgrounds. We have shown that both a multivalent pneumococcal conjugate vaccine covering all serotypes and a cross-protective common protein vaccine have the potential to significantly reduce the burden of pneumococcal disease in children under age 5 years. It is unlikely a vaccine covering all serotypes will be developed. Cross-protective common protein vaccines are currently being investigated as alternate or synergistic strategies to improve the coverage against a broader diversity of pneumococcal serotypes.

Developing countries in general, and the poorer populations within them specifically, account for the greatest burden of disease due to pneumonia globally. An effective vaccine distributed worldwide will reduce that burden, and if delivery is targeted at the poorest areas, the gap of inequity in health will also be reduced.

While both types of vaccine appear to score well overall and are likely to have a high impact on reduction of disease burden and equity, the experts were not very optimistic about the feasibility (answerability) of the PPV. However, given that it is unlikely to develop a low cost PCV covering all serotypes, it may be worthwhile focussing on developing a low cost PPV. A limiting factor is that the experts felt that the development cost of a PPV is unlikely to be low, so though we may eventually develop such a vaccine, it might not be affordable for resource-poor developing countries to introduce the vaccine without active support of international agencies like the GAVI Alliance.

One of the factors influencing efficacy estimates is the poor ability to actually identify the bacterial aetiology. Currently most of the aetiology-specific diagnosis is based on looking at reduction in pneumonia and "clinical or radiological signs". However this can be very confusing. For example, it is known that a proportion of children with RSV pneumonia will have clinical chest radiographs consistent with lobar pneumonia, which can be confused with a bacterial pneumonia, like pneumococcus [65]. Therefore, evaluation of diagnostics that do not require samples from within the lung, yet may be more sensitive than blood culture isolation, would be an aid to monitoring vaccine impact on IPD. These can be used inter-alia in studies estimating burden of disease studies as well as vaccine effectiveness and will help accurately interpret the impact of a vaccine.

This is the first time such an exercise has been attempted to predict the impact of emerging vaccines. CHNRI methodology was primarily designed to evaluate existing interventions and competing investment priorities for health research. Though we used the CHNRI criteria, we modified it by including systematic review of available literature and not involving all stakeholders (e.g. end-users and health workers). The scores included herewith express the collective opinion of a panel of 20 experts. There is always an element of uncertainty while predicting impact of interventions which do not exist and have no clinical trial data to support them. While we feel that the results would be reproducible with another panel of a similar composition in a different setting, this is a hypothesis that can be tested.

## Conclusions

To summarize, while it is not only important that investments are made in researching new vaccines, adequate emphasis must be made and resources allocated for proper distribution of the vaccine. Without adequate attention to these very real contextual factors and health systems issues, even the best investments can fail. Until that happens, we will see little reduction in the 800,000 child deaths per year due to pneumococcal pneumonia.

## Competing interests

The authors declare that they have no competing interests.

## Authors' contributions

All authors of this research paper have directly participated in the planning, execution, or analysis of the study and have read and approved the submitted version. In particular IR and HC designed the study and directed its implementation, including quality assurance and control. JW was responsible for the acquisition of the data and conducted the literature review. ET, ACS, HN, LZ, TH, HLJ, SM, CR, SEA, RK, TAJ and WAB helped design the study’s analytic strategy and prepared the Materials and Methods, Results and Discussion sections of the text. All authors of this research paper have critically revised the manuscript for important intellectual content.

## Supplementary Material

Additional file 1 Search Strategies Click here for file

Additional file 2 Questions used in the Phase II CHNRI process Click here for file

Additional file 3 The clinical trial process Click here for file
