# Supplementary material for: An evaluation of emerging vaccines for childhood pneumococcal pneumonia
Source: BMC Public Health. 2011 Apr 13;11(Suppl 3):S26. doi: 10.1186/1471-2458-11-S3-S26 (PMC3231900; doi:10.1186/1471-2458-11-S3-S26)
Supplement: Additional file 1 — Search Strategies [file 1471-2458-11-S3-S26-S1.doc]

# Additional File 1

**Additional File 1 - Search strategies**

Pneumococcal conjugate vaccines

| Number | Searches | Results |
| --- | --- | --- |
| 1 | Pneumococcal Vaccines/ | 2945 |
| 2 | pcv.ti,ab. | 2507 |
| 3 | 9-valent.mp. | 56 |
| 4 | nine-valent.mp. | 14 |
| 5 | 4 or 3 | 70 |
| 6 | 1 or 2 | 5328 |
| 7 | 6 and 5 | 42 |
| 8 | eleven-valent.mp. | 7 |
| 9 | 11-valent.mp. | 71 |
| 10 | exp clinical trial/ | 572961 |
| 11 | 10 and 5 | 23 |
| 12 | 8 or 9 | 72 |
| 13 | 10-valent.mp. | 9 |
| 14 | 6 and 13 | 4 |
| 15 | 13-valent.mp. | 18 |
| 16 | 6 and 15 | 12 |

Cross-protective common protein vaccine

| Number | Searches | Results |
| --- | --- | --- |
| 1 | vaccin$.ti,ab. | 150527 |
| 2 | vaccines/ or bacterial vaccines/ or vaccines, attenuated/ or vaccines, combined/ or vaccines, inactivated/ or vaccines, marker/ or vaccines, subunit/ or exp vaccines, synthetic/ or vaccines, conjugate/ | 47901 |
| 3 | 1 or 2 | 160000 |
| 4 | pneumonia, bacterial/ or pneumonia, pneumococcal/ or pneumonia, staphylococcal/ | 10390 |
| 5 | common protein.mp. | 459 |
| 6 | 3 and 5 | 14 |
| 7 | 4 and 6 | 2 |

Deliverability and equity

| Number | Searches | Results |
| --- | --- | --- |
| 1 | vaccin$.ti,ab. | 150527 |
| 2 | vaccines/ or bacterial vaccines/ or vaccines, attenuated/ or vaccines, combined/ or vaccines, inactivated/ or vaccines, marker/ or vaccines, subunit/ or exp vaccines, synthetic/ or vaccines, conjugate/ | 47901 |
| 3 | population characteristics/ or "health care facilities, manpower, and services"/ or "health care economics and organizations"/ or exp "costs and cost analysis"/ or economics, hospital/ or economics, medical/ or health care sector/ or health planning/ or state medicine/ | 218213 |
| 4 | cost$.ti,ab. | 226381 |
| 5 | deliver$.mp. | 315575 |
| 6 | deliver$.ti,ab. | 251177 |
| 7 | 1 or 2 | 160000 |
| 8 | 4 or 3 | 370347 |
| 9 | 6 or 5 | 315575 |
| 10 | 8 and 7 and 9 | 854 |
| 11 | pneumonia, bacterial/ or pneumonia, pneumococcal/ or pneumonia, staphylococcal/ | 10390 |
| 12 | EPI.mp. | 9120 |
| 13 | EPI.ti,ab. | 7991 |
| 14 | 13 or 12 | 9120 |
| 15 | 10 and 14 | 47 |

Global burden of disease

| Number | Searches | Results |
| --- | --- | --- |
| 1 | pneumonia, bacterial/ or pneumonia, pneumococcal/ or pneumonia, staphylococcal/ | 10390 |
| 2 | (global adj4 burden adj4 disease).mp. [mp=title, original title, abstract, name of substance word, subject heading word] | 459 |
| 3 | exp Pneumonia/ | 60468 |
| 4 | 2 and 3 | 1 |
| 5 | Streptococcus pneumoniae/ | 14803 |
| 6 | 5 and 2 | 2 |
| 7 | 1 and 2 | 1 |
